# Comparison of therapeutic effects of different acupuncture and moxibustion therapies on irritable bowel syndrome: A protocol for systematic review and network meta-analysis

**DOI:** 10.1097/MD.0000000000026920

**Published:** 2021-09-03

**Authors:** Jialei Guo, Li Yang, Jing He, Zhengming Yang

**Affiliations:** Lianyungang Hospital of Traditional Chinese Medicine, Lianyungang, Jiangsu Province, China.

**Keywords:** acupuncture and moxibustion, irritable bowel syndrome, network meta-analysis, protocol

## Abstract

**Background:**

Irritable bowel syndrome (IBS) is a functional gastrointestinal disorder with recurrent abdominal pain and changes in bowel habits. Many pieces of evidence show that acupuncture and moxibustion therapy has advantages in the treatment of IBS, but there are many acupuncture and moxibustion therapy options, each of which has different therapeutic effects. This study will evaluate the clinical efficacy of different acupuncture and moxibustion therapies in the treatment of IBS by means of a network meta-analysis.

**Methods:**

According to the retrieval strategy, we retrieved the randomized controlled trials of acupuncture and moxibustion treatment of IBS from China National Knowledge Infrastructure, Wanfang, VIP, Chinese biomedical databases, PubMed, Embase, Web of Science, and the Cochrane Library databases from the database establishment to July 2021. We assessed the quality of the studies using the Cochrane Risk Bias Assessment Tool and assessed the strength of the evidence using the Grading of Recommendation Assessment, Development, and Evaluation methodology. All data analyses were performed by RevMan5.3, Gemtc 0.14.3, and Stata 14.0.

**Results:**

This study evaluated the efficacy of different acupuncture and moxibustion therapies in the treatment of IBS by evaluating the clinical efficacy rate, symptom scores, quality of life scores, adverse reactions, etc, and further explore the mechanism of action of each therapy.

**Conclusion:**

This study will provide a reliable evidence-based basis for selecting the best acupuncture and moxibustion therapy for IBS.

**Ethics and dissemination:**

Private information from individuals will not be published. This systematic review also does not involve endangering participant rights. Ethical approval will not be required. The results may be published in a peer-reviewed journal or disseminated at relevant conferences.

**OSF Registration number:**

DOI 10.17605/OSF.IO/3278Y

## 1 Introduction

Irritable bowel syndrome (IBS) is a chronic functional bowel disorder characterized by abdominal pain and a change in bowel habits.^[1]^ It is estimated that IBS affects approximately 11.2% of the world's population, with 7.1% prevalence in North America^[2]^ and 5.9% in southeastern China.^[3]^ According to the Roman IV standard,^[4]^ patients with IBS could be divided into IBS with diarrhea (IBS-D), IBS with constipation, mixed IBS, and IBS unclassifiable, among which IBS-D was the most common subtype, accounting for about 40%.^[5]^ Although IBS does not cause organic damage to patients, its symptoms are recurrent. Long-term abdominal pain and abnormal defecation seriously harm patients’ physical and mental health and reduce their quality of life.^[6]^

The pathogenesis of IBS remains unclear, and treatment strategies focus on symptom management rather than disease improvement. Current treatments for IBS include lifestyle modifications, special diets, psychotherapy, and medication.^[7]^ Various medications are used to regulate problematic bowel habits or relieve abdominal pain, such as anticonvulsants, low-dose antidepressants, laxatives, and antidiarrheals.^[8]^ However, these drugs can only temporarily relieve the symptoms and have a high recurrence rate,^[9]^ and a large proportion of patients (60.1%) discontinue the drugs because they are not satisfied with the little improvement of symptoms.^[10]^ In addition, these drugs also have side effects such as headaches, dizziness, dry mouth, and insomnia, and serious adverse events such as cardiovascular disease and ischemic colitis may occur after long-term use.^[11]^

As a complementary and alternative therapy, acupuncture and moxibustion based on meridian theory have been widely used in IBS, with the characteristics of safety, effectiveness, and low cost.^[12]^ However, there are many forms of acupuncture and moxibustion therapies, such as needle acupuncture, electroacupuncture, moxibustion, and so on. Although they are all based on the meridian theory of traditional Chinese medicine, their use methods are different with different therapeutic effects. For example, moxibustion can improve the curative effect of IBS, improve its symptom score, and reduce inflammatory response compared with western medicine.^[13]^ Warm needle can effectively relieve symptoms, improve the quality of life, and reduce the recurrence rate compared with western medicine.^[14]^ Existing evidence shows that various forms of acupuncture and moxibustion therapies have advantages over western medicine in treating IBS. However, due to the lack of comparison between different forms of acupuncture and moxibustion therapies, it is not possible to judge which form of acupuncture and moxibustion therapy is better in treating IBS. Network meta-analysis is a method developed from traditional meta-analysis, which can compare the differences between 2 therapeutic measures through a common control when there is no direct comparison, so as to compare and rank the advantages and disadvantages of multiple clinical interventions.^[15]^ Therefore, this study uses the network meta-analysis method to compare the efficacy of different acupuncture and moxibustion therapies on IBS, so as to provide evidence for the selection of the optimal acupuncture and moxibustion treatment scheme in the clinical treatment of IBS.

## 2 Methods

### 2.1 Protocol register

This network meta-analysis was conducted according to the Preferred Reporting Items for Systematic Reviews and Meta-Analyses for Network Meta-Analysis guidelines.^[16]^ Moreover, it has been registered on open science framework (Registration number: DOI 10.17605/OSF.IO/3278Y).

### 2.2 Ethics

Since the protocol does not require patient recruitment and the collection of personal information, it does not require approval from an ethics committee.

### 2.3 Eligibility criteria

(1) Study object: patients clearly diagnosed with IBS (diagnostic criteria refer to Roman I–IV criteria ^[4,17]^), and gender and age were not limited.
(2) Study type: randomized controlled trials, not limited to blind, language limited to Chinese and English.
(3) Interventions: the treatment group was treated with conventional acupuncture, warm needle, electroacupuncture, fire acupuncture, moxibustion, auricular point sticking, acupoint embedding, and acupoint injection; the control group was treated with western medicine or placebo.
(4) Exclusion criteria
  i. For repeated publications, select the literature with the most complete data.
  ii. Studies in which the treatment group included traditional Chinese medicine treatments other than acupuncture-related therapies, such as Chinese medicine and massage.
  iii. Studies of which the patient was accompanied by severe intestinal diseases.
  iv. The type of publications were comments, experience presentations, conference articles, reviews, or case reports.

### 2.4 Outcome indicators

(1) Primary outcome indicators: clinical efficacy rate; symptoms scores (such as IBS-Symptom Severity Score^[18]^).
(2) Secondary outcome indicators: quality of life score (such as IBS-Quality of Life^[19]^), adverse reaction.

### 2.5 Search strategy

**Figure 1:**
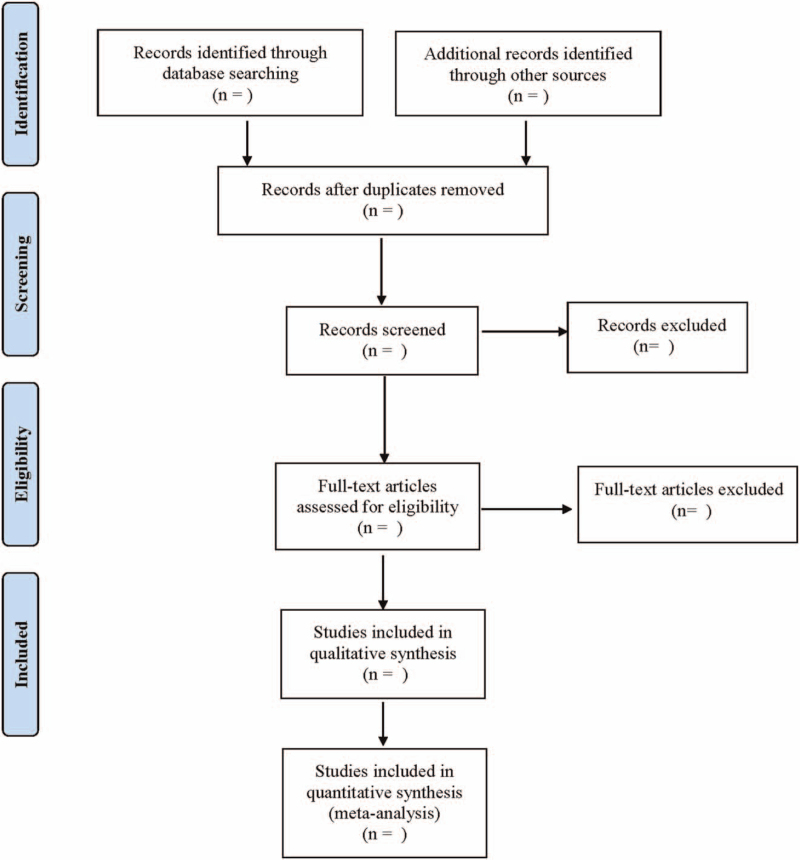
Flow diagram.

Two researchers independently searched from the establishment of the database to July 2021. Randomized controlled trials of different acupuncture and moxibustion therapies on IBS were searched by the computer on PubMed, Embase, Web of Science, Cochrane Library, China National Knowledge Infrastructure, Wanfang database, VIP, and Chinese biomedical databases. Chinese search terms were “zhen ci”(acupuncture), “dian zhen”(electroacupuncture), “wen zhen jiu”(warm needle), “huo zhen”(fire needle), “ai jiu”(moxibustion), “xue wei mai xian”(acupoint catgut embedding), “chang yi ji zong he zheng”(irritable bowel syndrome), etc. The English search terms were “acupuncture,” “electroacupuncture,” “warm needle,” “fire needle,” “moxibustion,” “acupoint catgut embedding,” “acupoint injection,” “Irritable Bowel Syndrome,” “IBS.” The included literature were independently screened by 2 researchers under the inclusion and exclusion criteria and decided in case of disagreement with the third researcher. The PubMed retrieval strategy is as shown in Figure 1.

### 2.6 Data screening and extraction

Literature screening and data extraction were independently and cross-checked by 2 researchers. Different agreements were discussed and decided with a third researcher. The information extracted were: the first author, year of publication, type of IBS, sample size, gender, age, course of IBS, study type, interventions, course of treatment, and outcome indicators. The literature screening process is shown in Table 1.

**Table 1:** Retrieval strategy of PubMed.

Number	Search terms
#1	Acupuncture [MeSH]
#2	Acupuncture [Title/Abstract]
#3	Pharmacopuncture [Title/Abstract]
#4	Electro-acupuncture [Title/Abstract]
#5	Warm needle [Title/Abstract]
#6	Fire needle [Title/Abstract]
#7	Blood-letting puncture [Title/Abstract]
#8	Moxibustion [MeSH]
#9	Moxibustion [Title/Abstract]
#10	Acupoint catgut embedding [Title/Abstract]
#11	#1 OR #2 OR #3 OR #4 OR #5 OR #6 OR #7 OR #8 OR #9 OR #10
#12	Irritable Bowel Syndromes [MeSH]
#13	Colon, Irritable [Title/Abstract]
#14	Colitis, Mucous [Title/Abstract]
#15	Mucous Colitides [Title/Abstract]
#16	#12 OR #13 OR #14 OR #15
#17	#16 AND #17

### 2.7 Literature quality assessment

Based on the tool used by the Cochrane Collaboration to assess the risk of bias in randomized trials, we assessed the risk of bias in the included literature in the following 7 aspects: (i) random sequence generation; (ii) allocation concealment; (iii) participant and personnel blinding; (iv) outcome assessment blinding; (v) incomplete outcome data; (vi) selective reporting; and (vii) other bias. The 2 researchers gave “low risk,” “high risk,” and “unclear” evaluations on the above content, and cross-checked the evaluation results. If there was a disagreement, and no agreement could be reached, the third researcher would discuss it. And finally, use RevMan5.3 to draw the bias risk map.

### 2.8 Statistical analysis

Stata14.0 software was used to draw an evidence network map to show the comparison of the intervention measures for each outcome indicator. GeMTC14.3 based on the Bayesian framework was used for network meta-analysis. The effect values of dichotomous variables were represented by the odds ratio, and the effective values of continuous variables were represented by mean difference. The 95% confidence interval was used to represent the statistical analysis results. A Markov Chain Monte Carlo fitting consistent model was used for Bayesian inference. Four chains were used for simulation, and the number of iterations was set as 50,000 (the first 20,000 for annealing and the last 30,000 for sampling). The potential scale reduction factor was used to reflect the convergence degree of the model. When the potential scale reduction factor was close to or equal to 1, it indicated that the data had good convergence and the obtained results were highly reliable.

### 2.9 Assessment of inconsistency

When there was a closed loop between the interventions, an inconsistency test was required. The *Z* test of STATA14.0 was used to evaluate the consistency of the results of direct comparison and indirect comparison. If *P* ≥ .05, it means that the possibility of inconsistency between direct comparison and indirect comparison is small. If *P* < .05, it means that there is a high possibility of inconsistency between direct comparison and indirect comparison, so fitting inconsistency analysis is needed. Calculate the surface under the cumulative ranking curve of different interventions through STATA 14.0. The larger surface under the cumulative ranking curve value was, the better the efficacy of the intervention. Finally, a comparison-correction diagram should be drawn to evaluate the existence of a small sample effect.

### 2.10 Sensitivity analysis

Given that studies with different levels of methodological quality may affect the final results, we conducted sensitivity analysis by excluding studies with a high risk of bias.

### 2.11 Assessment of publication bias

The comparison-adjusted funnel plots were obtained with the specific ranking order to detect small sample size study effects and publication bias. All analyses were conducted using R V.3.6.1 with the GeMTC package.

### 2.12 Evidence quality evaluation

Two investigators assessed the quality and recommended grading of all evidence of direct, indirect, and mixed estimates of all comparisons using a Grading of Recommendation Assessment, Development, and Evaluation methodology.^[20]^ The quality of evidence was rated on 4 levels: high, medium, low, or very low.

## 3 Discussion

IBS is a common and frequently occurring disease of the digestive system, and the number of visits is increasing year by year, which has always been one of the important research topics that medical workers pay attention to. The etiology and pathogenesis of IBS have not been fully clarified by modern medicine, and it is currently believed to be the result of the combined effects of many factors, such as gastrointestinal dynamics abnormality, visceral hypersensitivity, abnormal perception of the central nervous system to intestinal stimulation, intestinal infection and immune factors, intestinal microecological imbalance, and mental disorders.^[21]^

Acupuncture and moxibustion, a common external treatment derived from China, has the characteristics of multiple links, multiple levels, and multiple targets, with bidirectional regulating effects in the treatment of IBS.^[22]^ Different regulating effects can be generated by stimulating different types of peripheral nerve fibers.^[23]^ It has an obvious inhibitory effect on IBS-D intestinal motility, and can promote colon propulsion and relieve intestinal spasms on IBS with constipation. Different acupuncture and moxibustion therapies also have different mechanisms of action. For example, moxibustion can improve inflammatory response by inhibiting the role of IKKβ/IKBα/NF-κB signaling pathway.^[24]^ Electroacupuncture can reduce the levels of TNF-α and IL-6 to relieve visceral pain, regulate gastrointestinal contraction, and reduce inflammation.^[25]^ This study will explore the differences in the efficacy of different acupuncture and moxibustion therapies for IBS by means of network meta-analysis, and further explore the mechanism of action of different acupuncture and moxibustion therapies, so as to provide the evidence-based basis for clinical decision-makers to choose the optimal program.

However, our study still has some limitations: due to the limitations of language retrieval, we only included Chinese and English literature, which may cause selection bias; factors such as type, course of disease, and treatment of IBS may increase the possibility of heterogeneity. Nevertheless, we believe that the results of this research will help us to find the best acupuncture and moxibustion treatment scheme for IBS.

## Author contributions

**Data collection:** Jialei Guo, Li Yang

**Data curation:** Jialei Guo.

**Funding acquisition:** Zhengming Yang.

**Funding support:** Zhengming Yang

**Investigation:** Zhengming Yang.

**Resources:** Jialei Guo, Zhengming Yang, Li Yang.

**Software operating:** Jing He, Zhengming Yang

**Supervision:** Li Yang, Jing He

**Writing – original draft:** Jialei Guo, Li Yang

**Writing – review & editing:** Jialei Guo, Zhengming Yang, Li Yang.
